# Characterization of WY 14,643 and its Complex with Aldose Reductase

**DOI:** 10.1038/srep34394

**Published:** 2016-10-10

**Authors:** Michael R. Sawaya, Malkhey Verma, Vaishnavi Balendiran, Nigam P. Rath, Duilio Cascio, Ganesaratnam K. Balendiran

**Affiliations:** 1 UCLA-DOE, 611 Charles E. Young Drive East, 220 Boyer Hall, Los Angeles, CA 90095, USA; 2 Manchester Interdisciplinary Biocentre, 131 Princess Street, The University of Manchester, Manchester, M1 7DN, UK; 3 Department of Chemistry, WBSH 6017, Youngstown State University, One University Plaza, Youngstown, OH 44555, USA; 4 Department of Chemistry and Biochemistry, University of Missouri-St. Louis, St. Louis, MO 63121, USA.

## Abstract

The peroxisome proliferator, WY 14,643 exhibits a pure non-competitive inhibition pattern in the aldehyde reduction and in alcohol oxidation activities of human Aldose reductase (hAR). Fluorescence emission measurements of the equilibrium dissociation constants, K_d_, of oxidized (hAR•NADP^+^) and reduced (hAR•NADPH) holoenzyme complexes display a 2-fold difference between them. K_d_ values for the dissociation of WY 14,643 from the oxidized (hAR•NADP^+^•WY 14,643) and reduced (hAR•NADPH•WY 14,643) ternary complexes are comparable to each other. The ternary complex structure of hAR•NADP^+^•WY 14,643 reveals the first structural evidence of a fibrate class drug binding to hAR. These observations demonstrate how fibrate molecules such as WY 14,643, besides being valued as agonists for PPAR, also inhibit hAR.

Hyperlipidemia is a medical condition involving elevated levels of lipids in the blood, such as cholesterol and triglycerides. It causes blood vessels occlusion and increases the risk of developing atherosclerosis, coronary heart disease, strokes, hypertension or diabetes. According to March 2015 CDC report over 73.5 million adults (31.7%) in the United States have high low density lipoprotein, or “bad,” cholesterol. Clofibrate amphipathic carboxylic acids of a class known as fibrates, or peroxisome proliferators, are drugs once widely used in the clinical management of hyperlipidemia^1^. However, use was discontinued in the year 2002 due to its adverse effects of myopathy, myositis and rhabdomyolysis leading to acute renal failure. Pleiotropic response to fibrates, is characterized in the short term by upregulation of peroxisomal fatty acid P-oxidation enzymes and cytochrome P450 IVA in liver, peroxisomal proliferation, increased cell division and liver weight gain and in the longer term, pre-neoplastic lesions and eventually carcinomas of the liver. Clofibric acid glucuronide has been shown to mediate the formation of covalently bound clofibric acid-albumin adducts *in vitro*^2^ and clofibric acid-plasma protein adducts are identified in man and rat^3^. The covalent binding of drugs to tissue macromolecules has traditionally been associated with toxicity^4^,^5^,^6^,^7^.

Interestingly, a synthetic derivative of clofibrate, [4-Chloro-6-(2,3-xylidino)-2-pyrimidinylthio]acetic Acid (WY 14,643) (Fig. 1), is a potent anti hypercholesterolemic agent^8^. It is currently under investigation for prevention of heart failure resulting from hyperlipidemia. WY 14,643 has been shown to produce an 18-fold increased capacity in oxidization of palmitoyl-coenzyme A in rat livers^9^ and exert cardioprotection in a rat model of ischemia-reperfusion injury^10^. Also, WY 14,643 has been shown to improve metabolic indices, steatosis and ballooning in diabetic mice with non-alcoholic steatohepatitis^11^. A major mediator of WY 14,643 action is the ligand-activated transcription factor, peroxisome proliferator-activated receptor alpha (PPARα)^12^. Overall WY 14,643 is considered a potent murine (PPAR)α agonist and a weak PPARγ agonist^13^. Through this agonistic behavior, WY 14,643 increases PPAR transcriptional activity, thereby increasing levels of fatty acid oxidation, cell division, and cancer.

WY 14,643 has been known as a specific PPARα agonist since 1990. Only more recently, in 2006, this same inhibitor was also discovered to target Aldose reductase (hAR)^14^,^15^ and AKR1B10^16^, the Aldo-keto reductase protein family members. Inhibition kinetic data for WY 14,643 are *K*_ii_ (intercept inhibition constant) = *K*_is_ (the slope inhibition constant) = 1.8 μM and 1.67 μM and 1.63 μM for hAR catalyzed forward reaction and reverse reaction, respectively^15^. This finding suggested WY 14,643 follows a classical non-competitive pattern of inhibition with respect to the reduction of DL-glyceraldehyde and pure non-competitive mode in the oxidation of benzyl alcohol^15^. However molecular interactions, their corresponding binding affinity, stoichiometry, or location associated with hAR and WY 14,643 were yet to be revealed. AR catalyzed reduction of glucose to sorbitol is the first step of the polyol pathway through which less than 3% of the glucose flow in healthy human cells. However, under hyperglycemic or pre-diabetes conditions AR is overexpressed and the activity of this enzyme is implicated in the pathogenesis of most of the diabetic complications^17^,^18^,^19^,^20^,^21^. The sorbitol that is formed is not readily metabolized and leads to accumulation within the cell^22^,^23^. In addition, AR has also been linked in increased cardiovascular mortality rate with diabetic autonomic neuropathy patients^24^. These observations form the foundation to develop specific effective AR inhibitors.

We have determined the crystal structures of WY 14,643 and the hAR•NADP^+^•WY 14,643 ternary complex. Also we have applied fluorescence quenching methodologies to determine equilibrium dissociation constants binding of NADPH and NADP^+^ individually to apo hAR and binding of WY 14,643 to hAR•NADPH and hAR•NADP^+^ binary complexes separately. Here we report 1) these binding results, 2) protein free structure of WY 14,643, 3) hAR•NADP^+^•WY 14,643 ternary complex structure and describe conformational changes due to WY 14,643 binding to the hAR•NADP^+^ binary complex.

## Results

Fluorescence phenomenon seen by cofactor and inhibitor binding to hAR may be affected partly by charge transfer processes between groups/atoms. The characteristics of fluorescence reflect the outcome of the binding strength between the ligand and the protein that corresponds to the microenvironment and the conformational changes associated with hAR and other components.

### Fluorescence effect induced by cofactor and WY 14,643 binding

The fluorescence intensities of hAR decreased gradually when the concentrations of quenchers (i.e. inhibitor and cofactors) were increased. The observed fluorescence data were used for calculating the fluorescence quenching (Q% = (F^0^ − F)/F^0^) where F is the measured fluorescence and F^0^ is the fluorescence in the absence of quenchers.

**Figure 2:**
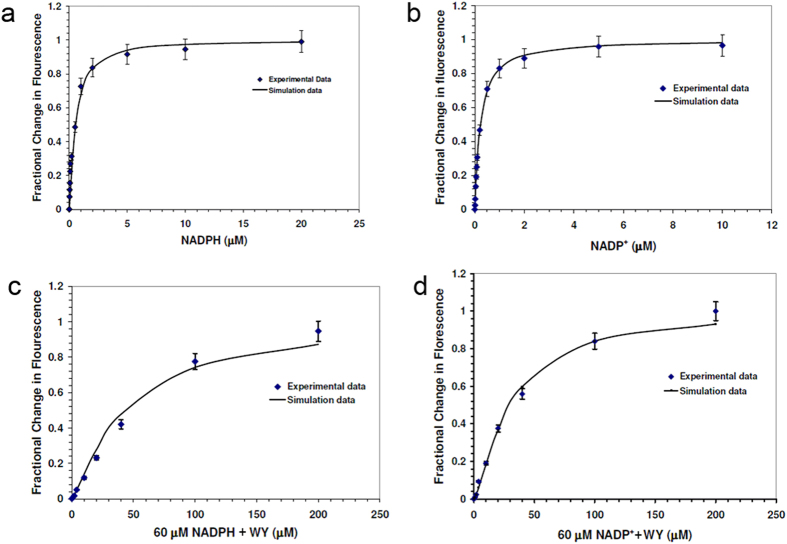
Fluorescence measurements for the determination of binding dissociation constant corresponding to (**a**). NADPH titration curve for hAR•NADPH complex formation (**b**). NADP^+^ titration curve for hAR•NADP^+^ complex formation (**c**). WY 14,643 titration curve for WY 14,643•hAR•NADPH complex formation and (**d**). WY 14,643 titration curve for WY 14,643•hAR•NADP^+^ complex formation, respectively.

The fractional change in fluorescence increased parabolically as the concentration of NADPH, NADP^+^ rose. The correlation between the average of triplicate measurements and simulated trend is shown in Fig. 2a,b (Table 1) for binding of NADPH to hAR and NADP^+^ to hAR, respectively. Our fluorescence emission measurements indicate that the equilibrium dissociation constants, K_d_s, are 0.210 μM and 0.48 μM for NADP^+^ and NADPH, respectively. Similar fractional changes in fluorescence followed a saturation pattern with increasing concentration of WY 14,643 in the presence of 60 μM NAPDH and NADP^+^ (Fig. 2c,d; Table 1). Furthermore the K_d_ values are very similar for WY 14,643 binding with hAR•NADP^+^ (0.104 μM) and hAR•NADPH (0.110 μM) binary complexes.

### Structure of hAR•NADP^+^•WY 14,643 Complex

The ternary complex structure, hAR•NADP^+^•WY 14,643, contains two protein molecules in the asymmetric unit (molA and molB). The two protein molecules are related by a non-crystallographic two-fold rotation axis. They superimpose with an RMS deviation of 0.07 Å over 316 pairs of Cα atoms. The ternary complex structure was refined to 1.65 Å resolution with the final R factor of 16.8%, and R_free_ of 19.5%. The structure contains a total of 632 protein residues corresponding to 316 amino acids for each protein molecule, two cofactors NADP^+^, two WY 14,643 molecules, 8 sulfate ions, and 569 water sites. The mean B values for the protein, cofactor, WY 14,643, sulfate and water atoms are 23.0, 14.6, 27.5, 49.6, and 33.9 Å^2^, respectively. Further crystallographic parameters are listed in Table 2.

### Atomic Interactions in hAR•NADP^+^•WY 14,643 Complex

Polar and vdW interactions stabilize an extended conformation of the cofactor. The detailed polar interactions between the cofactor and the protein atoms are shown in Table 3. One NADP^+^ molecule binds each molecule of the dimer of hAR in the ternary complex and the two cofactors in the asymmetric unit have an RMS deviation of 0.05 Å over 48 pairs of atoms.

**Figure 3:**
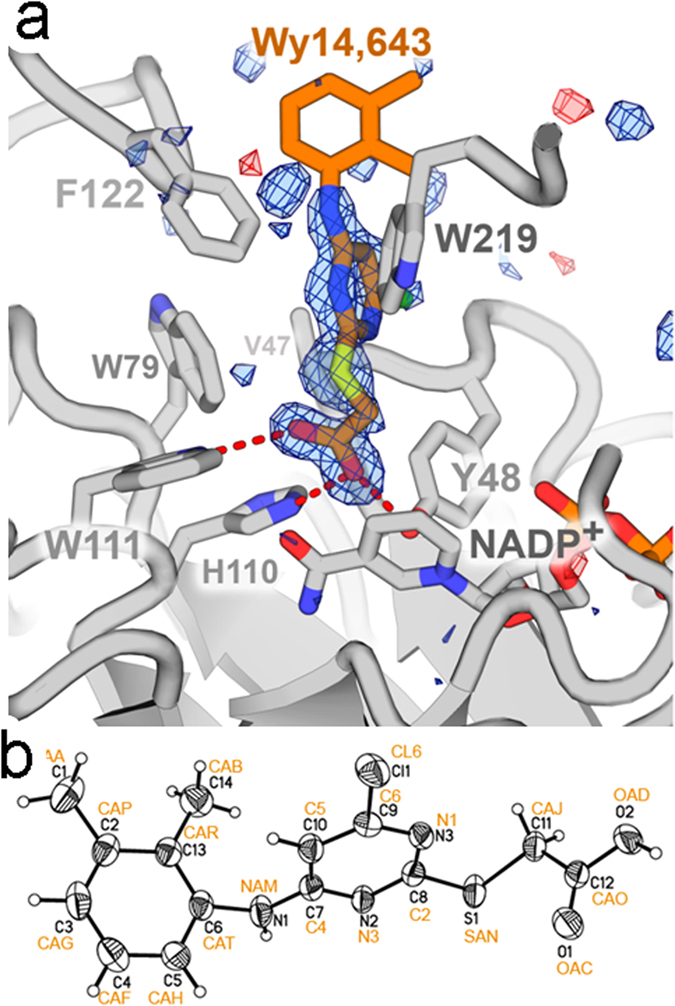
(**a**) Omit map corresponding to WY 14,643 in the hAR ternary complex. Simulated annealing refinement was performed on a copy of the coordinates omitting the inhibitor. The resulting Fo-Fc omit map is contoured at ±3.5 sigma (blue density is positive, red density is negative). In the refined structure, WY 14,643 is bound to hAR through a network of interactions O1•••OH of Tyr48, O1•••NE of His110, O2•••NE of His110, O2•••NE1 of Trp111, S•••SH of Cys298, S•••H of H_2_O381, N3•••NE1 of Trp20, N2•••H of H_2_O381. (**b**) Low temperature crystal structure of protein free WY 14,643 with thermal ellipsoid representation. Nomenclature adopted by the PDB V3 is shown in orange.

WY 14,643 binds hAR at the C-terminal opening of the α/β barrel, near the cofactor binding site (Fig. 3a). In our characterization of inhibitor binding, we divide the inhibitor into three regions, the carboxylate moiety, the pyrimidine moiety, and the 2,3-xylidino moiety. The carboxylate moiety of WY 14,643 anchors the inhibitor to hAR through three hydrogen bonds (Table 4) with Tyr48(OH), His110(NE2) and Trp111(NE1). It also makes a vdW contact with the nicotinamide ring, atom C3N (Fig. 3a; Table 4). Proximal to the carboxylate, the pyrimidine moiety of the inhibitor makes vdW contacts with the aromatic rings of Trp20, Trp219, and Phe122. Furthest from the carboxylate, the 2,3-xylidino moiety makes limited vdW contact with only one residue, Phe122. The density for this moiety is correspondingly weaker compared to the pyrimidine and carboxylate moieties. This evidence suggests that the two torsion angles between the pyrimidine and xylidino rings are free to rotate (*i.e*. rotate around the single bonds C4-NAM and NAM-CAT, nomenclature defined in Fig. 3b). We observe one molecule of WY 14,643 bound to each of the two hAR molecules. The RMS deviation between the two WY 14,643 molecules is 0.05 Å over 21 pairs of inhibitor atoms. Broken residual density at the 3.5 σ level suggests there may be an additional, lower occupancy, mode of binding of WY 14,643 in this same pocket.

**Figure 4:**
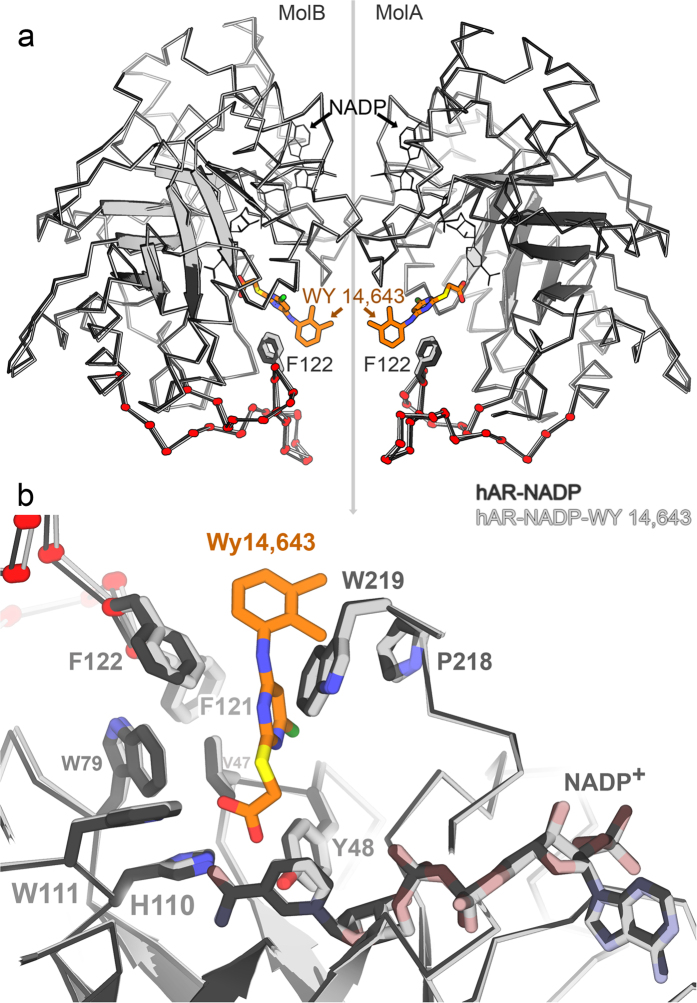
(**a**) Superposition of hAR binary hAR•NADP^+^ (PDB ID 3Q65) and ternary hAR•NADP^+^•WY 14,643 complexes. The Cα trace is shown for all atoms. Cartoon strands illustrate the orientation of the barrel. The binary complex is shown in light colors; the ternary complex is shown in dark colors. Overall, the structural differences between the binary and ternary complexes are very small. The red dots highlight the loop region 120–137 in which F122 (labeled) contacts the WY 14,643 (orange) 2,3-xylidino moiety and moves up to 0.5 Å. (**b**) Zoomed view around the active site of binary and ternary complex of hAR molA with NADP^+^ and WY 14,643. Residues are colored as in panel (a).

The conformation of the cofactor in the binary and ternary hAR complexes that is found in the dimeric crystal form is very similar. The RMS deviation is 0.25 Å between the dimer of hAR in the holoenzyme (PDB ID 3Q65) and the dimer of WY 14,643-bound ternary complex (624 pairs of Cα atoms). Notably, in the ternary hAR complex, the loop from residue 114 to 137 shifts closer to the 2,3-xylidino moiety of the inhibitor (Fig. 4a,b) in both molA and molB compared to the binary complex crystallized in the same space group. The RMS deviation corresponding to this loop (258 to 258 atoms) alone is 0.47 Å between the binary and ternary hAR complexes.

**Figure 5:**
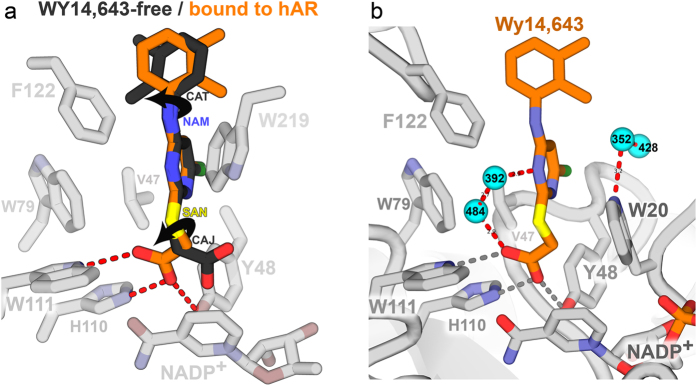
(**a**) Superposition of free and hAR-bound conformations of the WY 14,643 molecule. The two conformations are superimposed using only the atoms in their the pyrimidine rings. Arrows show the two torsion angles that rotate ~180° upon binding hAR in the ternary complex. (**b**) Waters (cyan spheres) stabilized by the presence of the WY 14,643 ligand in molA of hAR•NADP^+^•WY 14,643. The waters shown here are particular to the ternary complex and not observed in the hAR•NADP^+^ binary complex (PDB ID 3Q65). The corresponding water molecules in molB are W390, W386, W396, and W553, respectively.

The WY 14,643 inhibitor itself undergoes striking conformational changes upon binding to hAR. Comparison of WY 14,643 conformations in the protein free (Fig. 3b; Table 5) and hAR ternary complex structure reveals large deviations in two torsion angles (Fig. 5a,b). The first deviation is near the carboxylate moiety, involving ~180° rotation around the SAN-CAJ bond. This rotation is required to accommodate the hydrogen bonding geometry of the carboxylate with Tyr 48, His110, and Trp111 side chains and simultaneously avoid the clash between the pyridinium ring Cl atom and the side chain of Val47. The second deviation involves a ~180° rotation between the two aromatic rings, around the CAT-NAM bond. The rotation avoids collision between the methyl groups of WY 14,643 and the side chain atoms of Phe122. Due to the two flips, the overall the RMS deviation is large (1.7 Å) for the 21 pairs of inhibitor atoms corresponding to the protein free and hAR bound complex.

Binding of WY 14,643 does not perturb the core of the hAR active site especially near anion binding vicinity, but causes small structural changes in a loop proximal to the active site location. Several hydrophobic and other residues Trp20, Val47, Tyr48, His110, Trp111, Phe121, Phe122, Pro218, Trp219, Cys298 of hAR and nicotinamide ring of NADP^+^ 318 encompass the WY 14,643 binding site. Upon WY 14,643 binding to the holoenzyme, much of molA remains unperturbed, as evidenced by an RMS deviation of 0.12 Å over 312 pairs of Cα atoms; residues Trp20, Val47, Tyr48, His110, Trp111, Pro218, Trp219, Cys298 and nicotinamide ring of NADP^+^ 318 show no substantial conformational change. However residues Phe121 and Phe122 show the most noticeable structural movement, about 0.5 Å (Fig. 4b). Interestingly, residue Phe122 undergoes a conformational change with the corresponding RMS deviation of 0.67 Å over its 11 atoms. This change causes the adjacent residue Phe121 to move its side chain as well. The conformational changes that residues Phe122 and Phe121 undergo make their side chains parallel to the 2,3-xylidino moiety of WY 14,643 in the ternary complex.

The RMS deviation is 0.09 Å between the 48 atoms of molA NADP^+^ and corresponding atoms in the WY 14,643 ternary complex. Movements of the atoms in NADP^+^ triggered by WY 14,643 binding are minor.

Although most of the bound water molecules in the binary structure are present without significant changes in the ternary complex, we note additional water molecules form a network of hydrogen bonds between WY 14,643 and hAR in the ternary complex. Among the water molecules, some that occupy specific sites in the hAR binary complex, are still present in the WY 14,643 ternary complex almost around the same locations, but a few have shifted slightly. However, in the WY 14,643 ternary complex a distinct cluster of water molecules, W352, W392, W484, and W528 are situated around the polar atoms of the inhibitor of molA to form bridging interactions with hAR atoms (Fig. 5b). The corresponding water molecules in molB are W390, W386, W396, and W553, respectively.

### Comparison of hAR•NADP^+^•WY 14,643 complex with other ternary complexes

**Figure 6:**
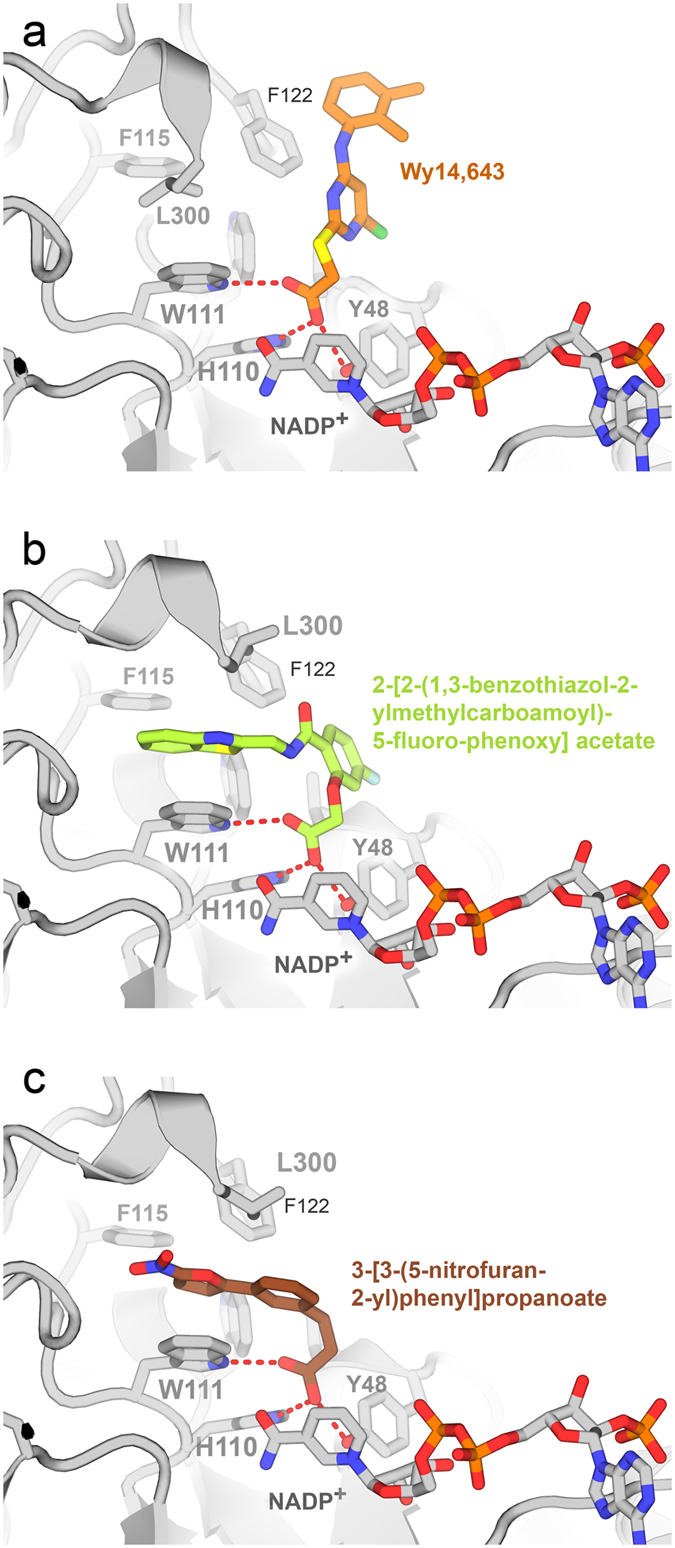
Comparison of binding geometries of WY 14,643 and two related ligands in the active site of hAR. Panel (a) shows WY 14,643 forms three hydrogen bonds between its carboxylate moeity and hAR (dashed red lines). These bonds are conserved among all thre analogs. Panel (b) shows 2-[2-(1,3-benzothiazol-2-ylmethylcarbamoyl)-5-fluoro-phenoxy]acetate bound to hAR (PDB code 4qr6). Note Leu300 moves to accommodate binding of its 1,3-benzothiazol ring. Panel (c) shows a similar movement in the hAR complex (PDB code 4prr) with 3-[3-(5-nitrofuran-2-yl)phenyl] propanoate.

There are several hAR inhibitor complex structures available but WY 14,643 is chemically divergent from all these inhibitors. Structures of PDB entries 4prr and 4qr6 of hAR ternary complexes with inhibitors 3-[3-(5-nitrofuran-2-yl)phenyl]propanoic acid and 2-[2-(1,3-benzothiazol-2-ylmethylcarbamoyl)-5-fluoro-phenoxy]acetic acid were compared with the WY 14,643 complex structure because these inhibitors partially share in common the presence of a carboxylate moiety with a connector atom (CH2, O, S) linking it to an aromatic ring by the same number of bonds. These complex structures superimpose RMSDs of 0.26 Å and 0.23 Å over 247 and 251 CA atoms of the WY 14,643 complex structure. However residues Phe115, Phe121-Val130, Ala299-Ser302 have shifted in WY 14,643 compared to these complexes. The carboxyl moiety of all these three inhibitors make very similar interactions with same residues of hAR but the phenoxy ring of 4qr6 overlaps with pyridine of WY 14,643 more than the phenyl group of 4prr (Fig. 6). Nevertheless furan and benzothiazol groups of 4prr and 4qr6 collide into the Leu300 as found in the WY 14,643 structure. The shift seen in the loop 299–302 between these complexes may be to accommodate different inhibitors in the 4prr and 4qr6 parallel to Tyr111 and perpendicular to Phe122 whereas in WY 14,643 perpendicular to Tyr111 and parallel to Phe122. This is because the bend around the CH2/O/S is different and phenyl/fluorophenoxy/pyrimidinyl groups and the rest of the inhibitors occupy different sites of the cavity/pocket than WY 14,643 in the hAR complexes.

## Discussion

Fluorescence spectroscopy is one of the most sensitive methods for studying structural changes in molecules. It can provide important information about (1) the overall conformation, (2) the presence of ligands, cofactors, substrates or inhibitors and (3) the interactions with and about intramolecular distances between specific chromophoric groups. For this reason the knowledge of the fluorescence properties is important in studies of structure-binding-function relationships. Quenching reactions are particularly key in this respect^25^ to follow binding events. Quenching of protein fluorescence by external quenchers is a useful technique for understanding the extent of exposure of the aromatic, side chains and to characterize their microenvironment^26^ especially to evaluate non-polar/vdW type interactions. As a result a variety of fluorescence quenching methodologies have been established^27^,^28^ to study ligand binding to biological molecules.

Current fluorescence quenching data imply oxidized cofactor NADP^+^ binds hAR with 2-fold higher affinity than the reduced cofactor NADPH. The difference between the oxidized and reduced forms of the cofactor resides in the nicotinamide ring which is aromatic and nonaromatic, respectively. The difference in planarity/electronics between the nicotinamide rings of the oxidized and reduced cofactors may be the major cause for the 2-fold difference reflected in the current measurements. The microenvironment surrounding the nicotinamide ring binding pocket might fit the ring in two unalike oxidation states differently. Hence the alterations in their interactions are reflected in the fluorescence signals.

Depending on the inhibitor used, inhibition patterns against aldehyde substrate for inhibition of AR are observed to be either uncompetitive or non-competitive. These observed inhibition patterns may imply the following scenarios that not all ARI bind at the enzyme active site, that the conformational change associated with nucleotide exchange is responsible for the rate determining step, that ARIs bind to both *E•NADP^+^ and *E•NADPH, that competition between substrate and inhibitor is masked in the overall rate of the reaction, or that tight binding causes non-competitive inhibition pattern.

Though the differences in pH, concentrations and different types of buffers may contribute to the disparity seen in the values/patterns of the parameters under consideration, employing fixed but different NADPH concentrations (0.15 mM compared to 60 μM) with different protein concentrations (0.5 mM versus 0.5 μM) will make significant variations observed by the above two methods. Furthermore 3-APADP^+^ used in the kinetic studies is chemically diverse with different affinity than NADP^+^ which is used in the quenching studies as 3-APADP^+^ is not currently commercially readily available. Therefore as Dr. Grimshaw demonstrated^29^ hAR•NADP^+^•WY 14,643 complex is anticipated to show significant inhibition of the steady-state turnover rate. The binding location of WY 14,643 in the active site as revealed by this ternary hAR complex structure reinforces such functional phenomenon.

The non-selective kinase inhibitor, staurosporine is an ATP non-competitive inhibitor of protein kinase C^30^ but the crystal structure of its complex with another form of protein kinase C^31^ as well as with protein kinase A^32^,^33^ indicates binding in the ATP-binding pocket. Also the tyrphostin inhibitor PP1, is an ATP non-competitive inhibitor of pp60^c−src ^34 however it is shown to bind to the ATP-binding pocket of another kinase, Hck^35^. Similarly the structure reveals binding of WY 14,643 in the active site of the hAR ternary complex.

Fluorescence data show WY 14,643 binds hAR•NADP^+^ as well as hAR•NADPH binary complexes with almost equal affinity. The x-ray crystal structure reported here confirms the binding of WY 14,643 to the hAR•NADP^+^ binary complex. In addition the structure of the hAR•NADP^+^•inhibitor ternary complex reveals small conformational changes associated with residues Phe122 and Phe121 upon WY 14,643 binding to hAR•NADP^+^ the binary complex. The carboxylate moiety of WY 14,643 interacts with polar atoms of His, Tyr, Trp and NAPD^+^ that have aromatic character and is surrounded by other side chains that are aromatic too. However these residues do not demonstrate significant conformational or positional changes upon binding of the inhibitor’s carboxyl moiety; large conformational changes would likely interfere with the long range Π interactions across this pocket. Therefore the majority of the differences reported by the fluorescence titration may originate from the microenvironment. Overall the current observations reinforce interpretations from previous kinetic data that WY 14,643 targets the hAR active site selectively in the presence of oxidized as well as reduced cofactors, NADP^+^ and NADPH.

**Figure 7:**
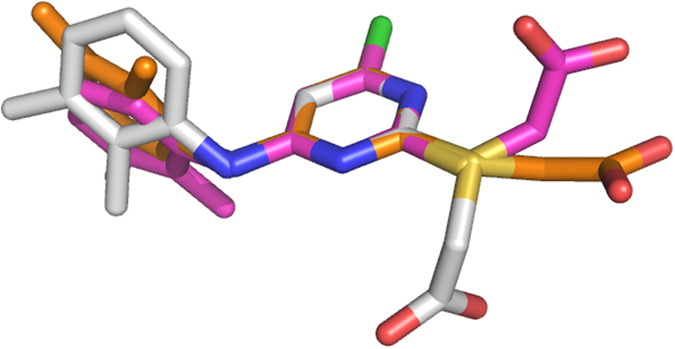
Superposition of free and PPAR bound conformations of WY 14,643. Conformations of WY 14,643, B1468 (white) B1470 (brown) bound to PPARα are superimposed using only the atoms in their the pyrimidine rings of protein free WY 14,643 (purple).

WY 14,643 with its strong hypolipidemic effects is known to be an agonist of PPARα and an inhibitor of hAR; structural evidence suggests that these two biological roles are facilitated by different conformations of this molecule. As reported (PDB entry 4BCR)^36^ WY 14,643 binds to two types of sites on PPARα–an active site and a secondary site. Since PPARα was observed in homodimeric form, there are two examples of each site: molecules labeled A1468 and B1468 bind to the agonist pocket and are fully buried by the protein, and molecules labeled A1469 and B1470 bind to secondary sites which are partially exposed to solvent. The conformation of WY 14,643 differs between agonist and secondary sites, varying by the same torsion angles that were found to differ between free and hAR-bound inhibitor molecules (Fig. 7) and by similar magnitudes. The variation can be explained by the large difference between the structure of the agonist and secondary sites. In the agonist pocket, WY 14,643 is bound through all three of its functional moieties with numerous polar and nonpolar contacts, whereas in the secondary site, the carboxylate moiety is mostly solvent exposed with only a single polar contact less than 3.4 Å (Table 4). Moreover, these two PPARα-bound conformations of WY 14,643 differ from the hAR-bound and free inhibitor conformations. The PPARα agonist pocket and secondary site conformations (21 atoms) superimpose with RMSD of 1.5 Å and 1.3 Å with the hAR•NADP^+^•WY 14,643 complex and 1.7 Å and 1.8 Å with free WY 14,643, respectively.

Administration of WY 14643 has been shown to protect against cardiomyocyte apoptosis following ischemia/reperfusion or biomechanical stress in the mouse heart^37^. In patients with type 2 diabetes, myocardial energetic status index (phosphocreatine-to-ATP ratio) negatively correlates with plasma free fatty acid (FFA) concentrations^38^. Diabetic patients have abnormal cardiac energy metabolism associated with high FFA concentrations. Direct energy-dissipating methods such as reduction of fat accumulation in adipocytes, or alteration of fatty acid metabolism, could be used to improve insulin resistance in type 2 diabetic patients^39^. In type 2 diabetes and even in individuals with family history of diabetes, mitochondrial metabolism, ATP synthesis are reduced in concert with a reduction of key factors regulating mitochondrial biogenesis, including amino acid biosynthesis and fatty acid oxidation^40^,^41^,^42^. Findings reported in the current study along with the information in the literature imply hAR is a target for WY 14,643 mechanism of action. Therefore the hAR mediated actions may contribute to the physiological outcomes of the clinical application of WY 14,643. Besides results from current binding and structure determination demonstrate that only one molecule of WY 14,643 binds to hAR and its carboxylate moiety occupies in the anion binding pocket under experimental conditions explored.

## Methods

### Production of recombinant hAR

His-tagged recombinant hAR was expressed in *E. coli* BL21 cells that were grown in Luria-Bertani broth containing 50 mg/L ampicillin with constant shaking in rotary shaker to reach the OD_600_ = 0.6–0.8 at 37 °C and 240 rpm. The protein expression was induced by supplementing 1 mM isopropyl-1-thio-galactopyranoside (IPTG) in the culture medium. The cells were harvested after 3–4 hrs by centrifugation (6000 *g*, 10 min) and resuspended in 50 mM sodium phosphate buffer (pH 7.0) containing 300 mM NaCl and 1 mM 2-mercaptoethanol and lysed by ultra-sonication. The hAR was isolated from the lysate separated by centrifugation at 10,000 g for 1.0 hr at 4 °C. The supernatant containing hexa-His-hAR was incubated for 1–2 hr by constant gentle mixing with Talon metal affinity matrix (Clontech, Mountain View, USA), later matrix slurry was passed through column and washed with 50 mM sodium phosphate buffer (pH 7.0) having 300 mM NaCl and 1.0 mM 2-mercaptoethanol. The protein was eluted with 150 mM imidazole in 50 mM sodium phosphate buffer (pH 7.0) containing 300 mM NaCl and 1 mM 2-mercaptoethanol and dialyzed in the 50 mM sodium phosphate buffer (pH 7.0) containing 1 mM 2-mercaptoethanol. The His-tag was removed by thrombin cleavage (Novagen, USA) as per manufacturer’s instructions. hAR was further purified by anion exchange on DEAE Sephadex A25 column by binding with DEAE Sephadex A 25 matrix. The concentration of hAR was determined by the Bradford assay (Bio-Rad, Hercules, USA), the purity was assessed by SDS-PAGE and the enzyme activity was determined by using 10 mM DL-glyceraldehyde and 0.15 mM NADPH as substrate and cofactor respectively.

### Fluorescence titration for the binding of cofactors and inhibitors to hAR

Fluorescence titration was performed for the binding of NADPH to hAR at a protein concentration of 0.5 μM in 5 mM sodium phosphate buffer (pH 7.0) containing 100 μM DTT. The titration for NADP^+^ binding to hAR was conducted at a protein concentration of 0.5 μM in 10 mM Tris-HCl buffer (pH 9.0) containing 100 μM DTT in 1.4 ml quartz cuvette by measuring fluorescence by SpectraMax M2 (Molecular Device, CA). The concentration of protein•NADP^+^/NADPH complexes formed were measured by extensive quenching of protein fluorescence by varying the concentrations of NADP^+^/NADPH at excitation and emission wavelengths of 295 and 365 nm, respectively following the procedure described by^43^,^44^. This method was extended for inhibitor (WY 14,643) binding to enzyme•NADPH complex at protein concentration of 0.5 μM in 5 mM sodium phosphate buffer (pH 7.0) containing 100 μM DTT by keeping saturated fixed 60 μM concentration of NADPH and by varying the concentrations of WY 14,643. The binding of inhibitor (WY 14,643) to enzyme•NADP^+^ complex was measured at 0.5 μM protein concentration in 10 mM Tris-HCl buffer (pH 9.0) containing 100 μM DTT by keeping saturated fixed 60 μM concentration of NADP^+^ and varying the WY 14,643. The concentrations of enzyme•NADP^+^/NADPH•inhibitor complex formed were measured by quenching of fluorescence protein•NADP^+^/NADPH complexes with excitation and emission wavelengths 295 and 365 nm, respectively.

### Determination of binding dissociation constants and stoichiometric coefficients of cofactors and WY 14,643 to hAR using fluorescence titration data

The methodology described by van de Weert 2010 was followed in the binding constant determination^27^,^28^. The fractional saturation (α) of protein by cofactors (NADP^+^/NADPH) and by inhibitor (WY 14,643) binding to protein can be expressed in the form of fluorescence as follows;


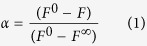


where *F*^0^ is the fluorescence in absence of quenchers (cofactors and inhibitor) denoted by L. *F* is the fluorescence at a given quencher concentration and *F*^∞^ is the fluorescence from a protein “saturated” of quencher. Assuming that quenchers bind to protein with molar stoichiometry of 1:n as shown in the equation (2);


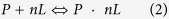


where n represents the order (stoichiometry) of kinetic reaction, *k*_+1_ and *k*_−1_ represent binding and dissociation rate constants respectively. The equilibrium dissociation constant can be expressed as below;


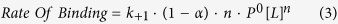



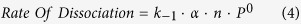


At equilibrium;
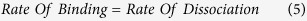



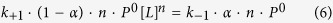


The equilibrium dissociation constant can be expressed as below;


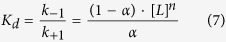


This equation can be re-arranged to,


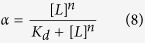


when quencher concentration (L) ≈ Initial concentration (L^0^) then equation (8) can be expressed as follows;


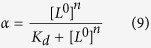


The binding dissociation constants and stoichiometry for the binding of quenchers (cofactors and inhibitor) to protein were calculated by non-linear fitting to the experimental fluorescence data and results are shown in Table 1.

### X-ray crystal structure determination of protein free WY 14,643

Crystals suitable for structure determination were obtained by crystallization of 4-Chloro-6-(2,3-xylidino)-2-pyrimidinylthioacetic acid (also called WY 14,643 or Pirinixic acid) from its solution in aqueous ethanol at room temperature. A single crystal with dimensions 0.21 × 0.21 × 0.21 mm^3^ was mounted on a glass fiber in a random orientation. Initial examination and data collection were performed using a Bruker APEX II Charge Coupled Device (CCD) Detector single crystal x-ray diffractometer using graphite monochromated Mo Kα radiation (λ = 0.71073 Å). Preliminary unit cell constants were determined with a set of 36 narrow frames. Intensity data were collected using ϖ and ϕ scans at a crystal to detector distance of 4.00 cm. The collected frames were integrated using an orientation matrix determined from the narrow frame scans. Bruker Apex2 and SAINT software packages^45^ were used for data collection and integration. Final unit cell constants were determined by global refinement of xyz centroids of threshold reflections from the complete data set. Collected data were corrected for systematic errors using SADABS^46^ based on the Laue symmetry using equivalent reflections. Crystal data and intensity data collection parameters for WY 14,643 obtained at low temperature are listed in Table 5.

Structure solution and refinement were carried out using the SHELXTL- PLUS software package^47^. The structure was determined by direct methods and refined successfully in the monoclinic space group, P 2_1_/c. Full matrix least-squares refinement was carried out by minimizing Σw(F_o_^2^ − F_c_^2^)^2^. The non-hydrogen atoms were refined anisotropically to convergence with final residual values: R1 = 3.2% and wR(F^2^) = 8.0%. All OH and NH hydrogen atoms were located from difference Fourier maps and were refined freely using isotropic thermal parameters like in the structure determination of fenofibric acid and fenofibrate following established procedure^47^,^48^,^49^. All other H atoms were treated using appropriate riding models (AFIX m3). Refinement parameters for the final structure of WY 14,643 are listed in Table 5. See supplementary information.

### Crystal structure determination of hAR with WY 14,643

Crystals of hAR•NADP^+^•WY 14,643 were generated using the hanging drop vapor diffusion method following a procedure that we previously established for the holoenzyme^50^. Protein, NADP^+^ and WY 14,643 solutions were mixed to achieve a molar ratio of 1:3:1.2 for the protein to cofactor to inhibitor. Crystals were briefly transferred to a solution containing the reservoir solution supplemented with 35% glycerol and flash-cooled by plunging them into liquid nitrogen. Data were collected at 100 K using beamline 9-2 at the Stanford Synchrotron Radiation Laboratory (SSRL) with an exposure time of 90 sec per 0.5° frame, a 250 mm crystal-to-detector distance, and wavelength of 0.9795 Å. The beamline was equipped with an ADSC Quantum 315 CCD detector. The data were processed and scaled to 1.8 Å resolution with XDS^51^, AIMLESS^52^,^53^ and AUTOPROC^54^ yielding an R_merge_ of 12.2%. The crystal belonged to space group P2_1_2_1_2_1_ and contained two hAR•NADP^+^•WY 14,643 complexes in the asymmetric unit. Initial phases were obtained by the difference Fourier method, using the holoenzyme (PDB ID 3Q65) (excluding solvent molecules) as the starting model. Initial rigid-body refinement and subsequent individual atomic refinement were performed using the programs REFMAC^53^,^55^ and BUSTER^56^. Model building was performed with the program COOT^57^. Clear electron density allowed the positioning of the WY 14,643 ligand and solvent molecules. The final model was validated with the following structure validation tools: PROCHECK^58^, ERRAT^59^ and VERIFY3D^60^. The Ramachandran plot indicates 91% of the residues lie in the most favoured regions and 9% of the residues lie in the additionally favoured regions. The ERRAT score was 96.7%. Details of the data collections and the refinement statistics are shown in Table 2.

## Additional Information

**How to cite this article**: Sawaya, M. R. *et al*. Characterization of WY 14,643 and its Complex with Aldose Reductase. *Sci. Rep.* **6**, 34394; doi: 10.1038/srep34394 (2016).

## Supplementary Material

Supplementary Information

## Figures and Tables

**Figure 1:**
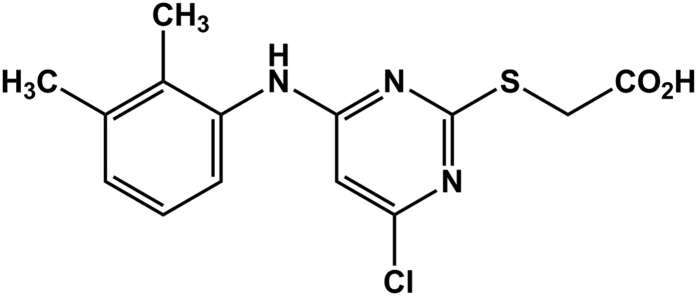
Chemical Structure of 4-Chloro-6-(2,3-xylidino)-2-pyrimidinylthioacetic acid, WY 14,643, Pirinixic acid.

**Table 1:** Equilibrium dissociation constants (K_d_) and stoichiometry coefficients (n) of NADP^+^ and NADPH binding with hAR and those of WY 14,643 binding with hAR•NADP^+^/NADPH complexes.

Quencher (L)	Complex	
	K_d(E-NADP_^+^) (μM)	K_d(E-NADPH)_ (μM)
NADP^+^	0.210 ± 0.01; n = 1.05 ± 0.03	
NADPH		0.48 ± 0.04; n = 1.25 ± 0.1
	**Complex**	
	**hAR•NADP**^**+**^	**hAR•NADPH**
WY 14,643	104.6 ± 4.2;n = 1.37 ± 0.05	110 ± 5.0;n = 1.25 ± 0.06

**Table 2:** Crystallographic Statistics of the hAR•NADP^+^•WY 14,643 complex structure.

Structures	hAR•NADP^+^•WY 14,643
Data collection, processing and structure refinement	
Wavelength (Å)	0.9795
Space group	P2_1_2_1_2_1_
Unit cell parameters	
a, b, c (Å)	83.3, 85.9, 104.4
α, β, γ (°)	α = β = γ = 90
Diffraction data	
Resolution range (Å)	100–1.65 (1.74–1.65)
Unique reflections	90,282 (12,715)
R_merge_ (%)	5.8 (45.2)
Completeness (%)	99.5 (97.2)
Redundancy	5.7 (3.9)
I/σ (I)	14.8 (2.5)
Refinement	
Resolution range used in refinement (Å)	66–1.65 (1.69–1.65)
Reflections used in refinement (work/free)	85,620 (4,556)
Final R values (work/free) (%)	16.8 (31.5)/19.5 (29.9)
Protein atoms	5,099
Cofactor atoms	96
Water molecules	569
Sulfate atoms	40
rmsd values	
Bonds (Å)	0.014
Angles (°)	1.7
PDB ID code	5HA7

**Table 3:** Distances between cofactor polar atoms and that of hAR or solvent.

NADP^+^ Atoms	dimeric hAR (ternary) complex			dimeric hAR (binary) complex		
	atom	residue	distance (Å)	atom	residue	distance (Å)
AOP1	OG	Ser263	2.7	OG	Ser263	2.67
	OG1	Thr265	2.7	OG1	Thr265	2.64
AOP2	NZ	Lys262	2.7	NZ	Lys262	2.63
	N	Val264	3.1			
AOP3	H	W135	2.6	H	W132	2.79
	H	W203	2.6	H	W49	2.54
AO2*	H	W135	3.0	H	W132	2.9
AO2*	NH1	Arg268	3.3	NH1	Arg268	3.18
AO3*	NE2	BGln26	3.0	OE1	BGln26	3.16
AO3*	H	W135	2.8	OD2	Asp216	3.12
AN7				ND2	Asn272	3.07
AN1				H	W35	2.55
AN6				OD1	Asn272	2.93
				OE2	Glu272	2.81
AO1	N	Ser214	3.1	N	Ser214	3.12
	N	Leu212	2.8	N	Leu212	2.86
AO2	N	Lys262	2.9	N	Lys262	2.97
NO1	NZ	Lys21	2.9	NZ	Lys21	2.83
NO2	OG	Ser210	2.9	OG	Ser210	2.76
	OG	Ser214	2.7			
NO5*	N	Ser210	3.2	N	Ser210	3.07
NO2*	OD2	Asp43	2.7	OD2	Asp43	2.58
NO3*	N	Trp20	2.9	N	Trp20	2.88
NO3*	N	Trp19	3.2	N	Thr19	3.11
NN7	OE1	Gln183	3.0	OE1	Gln183	3.09
	OG	Ser159	2.8	OG	Ser159	2.80
NO7	ND2	Asn160	2.8	ND2	Asn160	2.79

A-first molecule (molA); B-second molecule (molB) of the dimer; W-water molecule.

**Table 4:** Atomic distances between polar atoms of WY 14,643 and that in hAR ternary complex and PPAR complex.

WY 14,643Atom	dimeric hAR (ternary)			B1468•PPARα molB (4BCR)			A1469•PPARα molA (4BCR)		
	atom	residue	distance (Å)	atom	residue	distance (Å)	atom	residue	distance (Å)
O1	OH	Tyr48	2.9	OH	Tyr314	2.9	ND1	His274	2.6
	NE	His110	2.7	NE2	His440	3.5			
				OH	Tyr464	3.0			
O2	NE	His110	3.2	OH	Tyr314	3.1			
	NE1	Trp111	3.1	OG	Ser280	2.4			
S	SH	Cys298	3.9						
	H	W381	3.3						
N3	NE1	Trp20	3.2						
N2	H	W381	2.7	OG	Ser280	3.5			
N1				OG	Ser280	3.3			

W-water molecule; WY 14,643 atoms are labeled differently in hAR and PPARα structure coordinate set 4BCR. Atom labels matched in protein complexes hAR/4BCR-B1468/B4BCR-B1470 are C1/CAA/CAA, C2/CAP/CAP, C3/CAG/CAG, C4/CAF/CAF, C5/CAH/CAH, C6/CAT/CAT, C13/CAR/CAR, C14/CAB/CAB, N1, NAM/NAM, C7/C4/C4, N2/N3/N3, C8/C2/C2, N3/N1/N1, C9/C6/C6, C10/C5/C5, Cl/Cl6/Cl6, S/SAN/SAN, C11/CAJ/CAJ, C12/CAO, O1/OAD/OAD, O2/OAC/OAC.

**Table 5:** Crystal data and structure refinement for protein free WY 14,643.

Identification code	CCDC 1476501	
Empirical formula	C_14_H_14_ClN_3_O_2_S	
Formula weight	323.79	
Temperature	100(2) K	
Wavelength	0.71073 Å	
Crystal system	Monoclinic	
Space group	P 2_1_/c	
Unit cell dimensions	a = 7.1294(3) Å	α = 90°
	b = 22.6174(9) Å	β = 105.003(2)°
	c = 9.2174(4) Å	γ = 90°
Volume	1435.63(10) Å^3^	
Z	4	
Density (calculated)	1.498 Mg/m^3^	
Absorption coefficient	0.419 mm^−1^	
F (000)	672	
Crystal size	0.21 × 0.21 × 0.21 mm^3^	
Theta range for data collection	1.801 to 30.555°	
Index ranges	−9 ≤ h ≤ 10, −32 ≤ k ≤ 31, −13 ≤l ≤ 12	
Reflections collected	36,288	
Independent reflections	4,396 [R (int) = 0.0452]	
Completeness to theta = 25.242°	100.0%	
Absorption correction	Semi-empirical from equivalents	
Max. and min. transmission	0.8622 and 0.7643	
Refinement method	Full-matrix least-squares on F^2^	
Data/restraints/parameters	4,396/0/200	
Goodness-of-fit on F^2^	1.042	
Final R indices [I > 2sigma (I)]	R1 = 0.0321, wR2 = 0.0740	
R indices (all data)	R1 = 0.0427, wR2 = 0.0797	
Largest diff. peak and hole	0.451 and −0.296 e.Å^−3^	
