# Supplementary material for: Characterization of WY 14,643 and its Complex with Aldose Reductase
Source: Sci Rep. 2016 Oct 10;6:34394. doi: 10.1038/srep34394 (PMC5056380; doi:10.1038/srep34394)

Characterization of WY 14,643 and its Complex with Aldose Reductase

Michael Sawaya[1], Malkhey Verma[2‡], Vaishnavi Balendiran[3], Nigam P. Rath[4], Duilio Cascio[1],

Ganesaratnam K. Balendiran[3,*]

FW: CCDC Depository Request CRM:0001000398915 Pe

Dear Depositor,
Thank you for depositing your crystal structure(s) at the Cambridge Crystallographic Data Centre.
The data have been assigned to the following deposition numbers.
CCDC 1476501

Summary of Data CCDC 1476501

Compound Name:
Formula: C14 H14 Cl1 N3 O2 S1
Unit Cell Parameters: a 7.1294(3) b 22.6174(9) c 9.2174(4) P21/c

----

If we have any queries relating to the data then we will contact you later.
Data submitted as a CSD Communications (previously known as Private Communications) will be processed and added to the Cambridge Structural Database (CSD).
Please note, if the data have not appeared in a journal publication 1 year after the date of deposition,
and the CCDC cannot contact you to discuss the matter, then the CCDC will automatically include the data
in the CSD as a CSD Communications (previously known as Private Communications).
Kind regards,

The CSD Team

Cambridge Crystallographic Data Centre
http://www.ccdc.cam.ac.uk/

checkCIF/PLATON report

Structure factors have been supplied for datablock(s) r14213lt

THIS REPORT IS FOR GUIDANCE ONLY. IF USED AS PART OF A REVIEW PROCEDURE FOR PUBLICATION, IT SHOULD NOT REPLACE THE EXPERTISE OF AN EXPERIENCED CRYSTALLOGRAPHIC REFEREE.

No syntax errors found. CIF dictionary Interpreting this report

Datablock: r14213lt

```
Bond precision:  C-C = 0.0018 A          Wavelength=0.71073

Cell:            a=7.1294(3)     b=22.6174(9)        c=9.2174(4)
                 alpha=90        beta=105.003(2)     gamma=90
Temperature:     100 K

                Calculated                 Reported
Volume          1435.63(10)                1435.63(10)
Space group     P 21/c                     P 21/c
Hall group      -P 2ybc                    -P 2ybc
Moiety formula  C14 H14 Cl N3 O2 S         C14 H14 Cl N3 O2 S
Sum formula     C14 H14 Cl N3 O2 S         C14 H14 Cl N3 O2 S
Mr              323.80                     323.79
Dx,g cm-3       1.498                      1.498
Z               4                          4
Mu (mm-1)       0.419                      0.419
F000            672.0                      672.0
F000'           673.32
h,k,lmax        10,32,13                   10,32,13
Nref            4406                       4396
Tmin,Tmax       0.915,0.917                0.764,0.862
Tmin'           0.915

Correction method= MULTI-SCAN

Data completeness= 0.998          Theta(max)= 30.555

R(reflections)= 0.0321( 3670)     wR2(reflections)= 0.0797( 4396)

S = 1.042                  Npar= 200
```

The following ALERTS were generated. Each ALERT has the format
test-name_ALERT_alert-type_alert-level.
Click on the hyperlinks for more details of the test.

Alert level G

```
PLAT005_ALERT_5_G No _iucr_refine_instructions_details  in the CIF          ? Do !
PLAT912_ALERT_4_G Missing # of FCF Reflections Above STh/L=  0.600         10
```

```
   0 ALERT level A = Most likely a serious problem - resolve or explain
   0 ALERT level B = A potentially serious problem, consider carefully
   0 ALERT level C = Check. Ensure it is not caused by an omission or oversight
   2 ALERT level G = General information/check it is not something unexpected

   0 ALERT type 1 CIF construction/syntax error, inconsistent or missing data
   0 ALERT type 2 Indicator that the structure model may be wrong or deficient
   0 ALERT type 3 Indicator that the structure quality may be low
   1 ALERT type 4 Improvement, methodology, query or suggestion
   1 ALERT type 5 Informative message, check
```

It is advisable to attempt to resolve as many as possible of the alerts in all categories. Often the minor alerts point to easily fixed oversights, errors and omissions in your CIF or refinement strategy, so attention to these fine details can be worthwhile. In order to resolve some of the more serious problems it may be necessary to carry out additional measurements or structure refinements. However, the purpose of your study may justify the reported deviations and the more serious of these should normally be commented upon in the discussion or experimental section of a paper or in the "special_details" fields of the CIF. checkCIF was carefully designed to identify outliers and unusual parameters, but every test has its limitations and alerts that are not important in a particular case may appear. Conversely, the absence of alerts does not guarantee there are no aspects of the results needing attention. It is up to the individual to critically assess their own results and, if necessary, seek expert advice.

Publication of your CIF in IUCr journals

A basic structural check has been run on your CIF. These basic checks will be run on all CIFs submitted for publication in IUCr journals (*Acta Crystallographica*, *Journal of Applied Crystallography*, *Journal of Synchrotron Radiation*); however, if you intend to submit to *Acta Crystallographica Section C* or *E*, you should make sure that full publication checks are run on the final version of your CIF prior to submission.

Publication of your CIF in other journals

Please refer to the *Notes for Authors* of the relevant journal for any special instructions relating to CIF submission.

PLATON version of 01/06/2013; check.def file version of 24/05/2013

Datablock r14213lt - ellipsoid plot

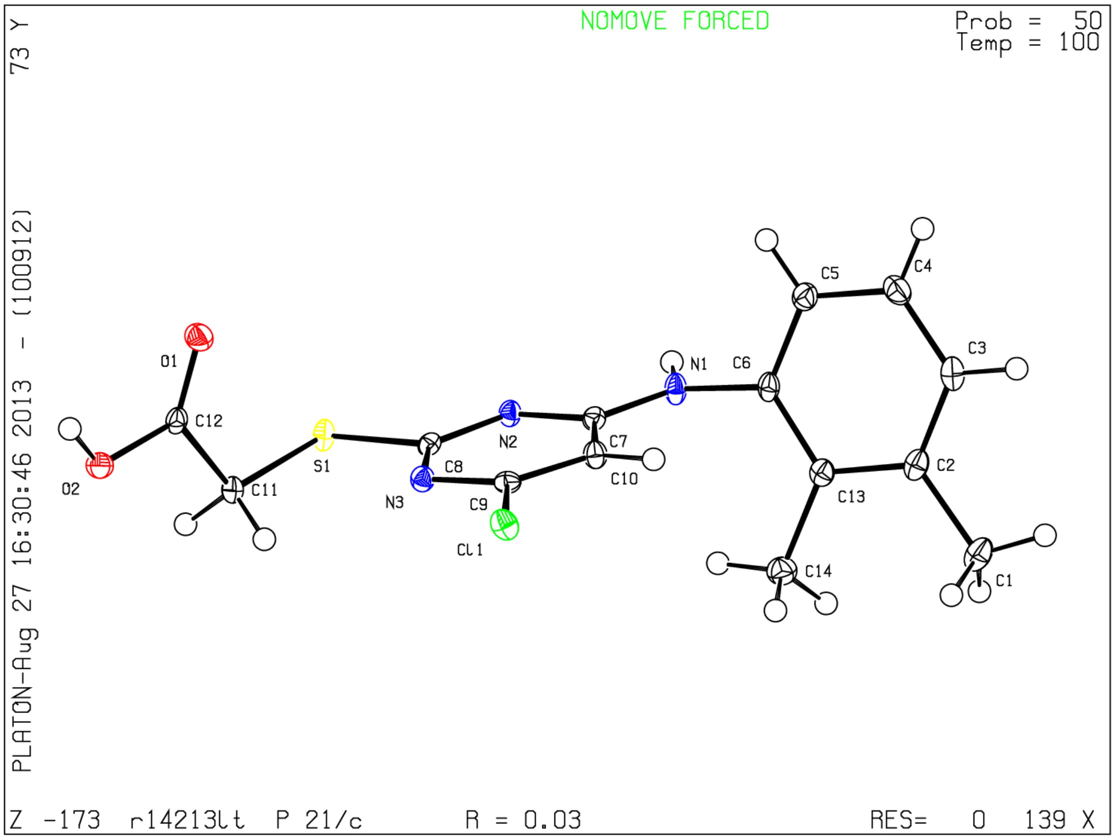

Table 1. Crystal data and structure refinement for npr14213-lt.

Identification code	r14213lt/x8/Bali/GKB529B	
Empirical formula	C14 H14 Cl N3 O2 S	
Formula weight	323.79	
Temperature	100(2) K	
Wavelength	0.71073 Å	
Crystal system	Monoclinic	
Space group	P 2_1/c	
Unit cell dimensions	a = 7.1294(3) Å	α= 90°.
	b = 22.6174(9) Å	β= 105.003(2)°.
	c = 9.2174(4) Å	γ = 90°.
Volume	1435.63(10) $Å^3$	
Z	4	
Density (calculated)	1.498 Mg/m^3	
Absorption coefficient	0.419 mm^{-1}	
F(000)	672	
Crystal size	0.211 x 0.209 x 0.208 mm^3	
Theta range for data collection	1.801 to 30.555°.	
Index ranges	$-9 \leq h \leq 10$, $-32 \leq k \leq 31$, $-13 \leq l \leq 12$	
Reflections collected	36288	
Independent reflections	4396 [R(int) = 0.0452]	
Completeness to theta = 25.242°	100.0 %	
Absorption correction	Semi-empirical from equivalents	
Max. and min. transmission	0.8622 and 0.7643	
Refinement method	Full-matrix least-squares on F^2	
Data / restraints / parameters	4396 / 0 / 200	
Goodness-of-fit on F^2	1.042	
Final R indices [I>2sigma(I)]	R1 = 0.0321, wR2 = 0.0740	
R indices (all data)	R1 = 0.0427, wR2 = 0.0797	
Extinction coefficient	n/a	
Largest diff. peak and hole	0.451 and -0.296 e.$Å^{-3}$	

Table 2. Atomic coordinates (x 10^4) and equivalent isotropic displacement parameters ($Å^2$x 10^3) for npr14213-lt. U(eq) is defined as one third of the trace of the orthogonalized U^{ij} tensor.

	x	y	z	U(eq)
Cl(1)	9956(1)	834(1)	9912(1)	15(1)
S(1)	5802(1)	2250(1)	12451(1)	14(1)
O(1)	9522(1)	2963(1)	12597(1)	17(1)
O(2)	11121(1)	2620(1)	14867(1)	16(1)
N(1)	2873(2)	1300(1)	7620(1)	14(1)
N(2)	4410(2)	1724(1)	9891(1)	12(1)
N(3)	7766(2)	1522(1)	11077(1)	12(1)
C(1)	2276(2)	-642(1)	4973(2)	18(1)
C(2)	2460(2)	22(1)	4992(2)	14(1)
C(3)	2342(2)	332(1)	3665(2)	16(1)
C(4)	2486(2)	943(1)	3654(2)	17(1)
C(5)	2710(2)	1255(1)	4976(2)	15(1)
C(6)	2790(2)	953(1)	6301(1)	12(1)
C(7)	4492(2)	1365(1)	8731(1)	11(1)
C(8)	6057(2)	1777(1)	10992(1)	11(1)
C(9)	7772(2)	1174(1)	9905(1)	11(1)
C(10)	6225(2)	1070(1)	8710(1)	13(1)
C(11)	8125(2)	2185(1)	13794(1)	13(1)
C(12)	9635(2)	2637(1)	13657(1)	12(1)
C(13)	2712(2)	335(1)	6349(1)	12(1)
C(14)	2868(2)	3(1)	7790(2)	17(1)

Table 3. Bond lengths [Å] and angles [°] for npr14213-lt.

Cl(1)-C(9)	1.7356(12)
S(1)-C(8)	1.7626(13)
S(1)-C(11)	1.7984(13)
O(1)-C(12)	1.2099(15)
O(2)-C(12)	1.3255(15)
O(2)-H(2)	0.85(2)
N(1)-C(7)	1.3383(16)
N(1)-C(6)	1.4349(16)
N(1)-H(1)	0.86(2)
N(2)-C(8)	1.3441(16)
N(2)-C(7)	1.3548(16)
N(3)-C(8)	1.3319(16)
N(3)-C(9)	1.3378(16)
C(1)-C(2)	1.5085(17)
C(1)-H(1A)	0.9800
C(1)-H(1B)	0.9800
C(1)-H(1C)	0.9800
C(2)-C(3)	1.3925(19)
C(2)-C(13)	1.4077(17)
C(3)-C(4)	1.3873(18)
C(3)-H(3)	0.9500
C(4)-C(5)	1.3819(18)
C(4)-H(4)	0.9500
C(5)-C(6)	1.3877(18)
C(5)-H(5)	0.9500
C(6)-C(13)	1.4009(17)
C(7)-C(10)	1.4084(17)
C(9)-C(10)	1.3625(17)
C(10)-H(10)	0.9500
C(11)-C(12)	1.5150(17)
C(11)-H(11A)	0.9900
C(11)-H(11B)	0.9900
C(13)-C(14)	1.5044(18)
C(14)-H(14A)	0.9800

C(14)-H(14B)	0.9800
C(14)-H(14C)	0.9800
C(8)-S(1)-C(11)	102.56(6)
C(12)-O(2)-H(2)	111.2(15)
C(7)-N(1)-C(6)	123.12(11)
C(7)-N(1)-H(1)	119.3(13)
C(6)-N(1)-H(1)	117.0(13)
C(8)-N(2)-C(7)	116.28(10)
C(8)-N(3)-C(9)	113.68(10)
C(2)-C(1)-H(1A)	109.5
C(2)-C(1)-H(1B)	109.5
H(1A)-C(1)-H(1B)	109.5
C(2)-C(1)-H(1C)	109.5
H(1A)-C(1)-H(1C)	109.5
H(1B)-C(1)-H(1C)	109.5
C(3)-C(2)-C(13)	119.56(11)
C(3)-C(2)-C(1)	120.32(12)
C(13)-C(2)-C(1)	120.10(12)
C(4)-C(3)-C(2)	121.29(12)
C(4)-C(3)-H(3)	119.4
C(2)-C(3)-H(3)	119.4
C(5)-C(4)-C(3)	119.67(12)
C(5)-C(4)-H(4)	120.2
C(3)-C(4)-H(4)	120.2
C(4)-C(5)-C(6)	119.61(12)
C(4)-C(5)-H(5)	120.2
C(6)-C(5)-H(5)	120.2
C(5)-C(6)-C(13)	121.73(12)
C(5)-C(6)-N(1)	117.39(11)
C(13)-C(6)-N(1)	120.83(12)
N(1)-C(7)-N(2)	117.68(11)
N(1)-C(7)-C(10)	121.56(11)
N(2)-C(7)-C(10)	120.75(11)
N(3)-C(8)-N(2)	127.72(11)
N(3)-C(8)-S(1)	119.39(9)

N(2)-C(8)-S(1)	112.89(9)
N(3)-C(9)-C(10)	125.70(11)
N(3)-C(9)-Cl(1)	116.65(9)
C(10)-C(9)-Cl(1)	117.65(9)
C(9)-C(10)-C(7)	115.87(11)
C(9)-C(10)-H(10)	122.1
C(7)-C(10)-H(10)	122.1
C(12)-C(11)-S(1)	115.79(9)
C(12)-C(11)-H(11A)	108.3
S(1)-C(11)-H(11A)	108.3
C(12)-C(11)-H(11B)	108.3
S(1)-C(11)-H(11B)	108.3
H(11A)-C(11)-H(11B)	107.4
O(1)-C(12)-O(2)	124.72(11)
O(1)-C(12)-C(11)	125.39(11)
O(2)-C(12)-C(11)	109.88(10)
C(6)-C(13)-C(2)	118.08(12)
C(6)-C(13)-C(14)	122.08(11)
C(2)-C(13)-C(14)	119.84(11)
C(13)-C(14)-H(14A)	109.5
C(13)-C(14)-H(14B)	109.5
H(14A)-C(14)-H(14B)	109.5
C(13)-C(14)-H(14C)	109.5
H(14A)-C(14)-H(14C)	109.5
H(14B)-C(14)-H(14C)	109.5

Symmetry transformations used to generate equivalent atoms:

Table 4. Anisotropic displacement parameters ($Å^2$x 10^3) for npr14213-lt. The anisotropic displacement factor exponent takes the form: $-2\pi^2[h^2 a^{*2}U^{11} + ... + 2 h k a^* b^* U^{12}]$

	U^{11}	U^{22}	U^{33}	U^{23}	U^{13}	U^{12}
Cl(1)	11(1)	19(1)	15(1)	-1(1)	2(1)	4(1)
S(1)	11(1)	15(1)	14(1)	-6(1)	1(1)	0(1)
O(1)	16(1)	18(1)	15(1)	2(1)	2(1)	-3(1)
O(2)	13(1)	16(1)	17(1)	3(1)	-2(1)	-4(1)
N(1)	10(1)	15(1)	14(1)	-5(1)	0(1)	2(1)
N(2)	10(1)	12(1)	12(1)	-2(1)	1(1)	0(1)
N(3)	11(1)	12(1)	12(1)	-1(1)	2(1)	0(1)
C(1)	16(1)	14(1)	25(1)	-5(1)	4(1)	0(1)
C(2)	9(1)	14(1)	18(1)	-4(1)	2(1)	0(1)
C(3)	13(1)	19(1)	15(1)	-5(1)	2(1)	0(1)
C(4)	16(1)	20(1)	13(1)	1(1)	1(1)	0(1)
C(5)	13(1)	13(1)	16(1)	-1(1)	-1(1)	0(1)
C(6)	9(1)	14(1)	13(1)	-4(1)	0(1)	1(1)
C(7)	11(1)	10(1)	12(1)	0(1)	2(1)	-1(1)
C(8)	12(1)	9(1)	13(1)	0(1)	3(1)	-2(1)
C(9)	10(1)	11(1)	13(1)	2(1)	3(1)	1(1)
C(10)	12(1)	14(1)	12(1)	-2(1)	3(1)	1(1)
C(11)	13(1)	13(1)	12(1)	-1(1)	0(1)	-2(1)
C(12)	12(1)	10(1)	12(1)	-3(1)	2(1)	1(1)
C(13)	8(1)	14(1)	15(1)	-1(1)	1(1)	0(1)
C(14)	17(1)	17(1)	18(1)	2(1)	4(1)	-1(1)

Table 5. Hydrogen coordinates (x 10^4) and isotropic displacement parameters (Å^{2}x 10^3) for npr14213-lt.

	x	y	z	U(eq)
H(1A)	1888	-782	3931	28
H(1B)	1291	-759	5488	28
H(1C)	3527	-818	5486	28
H(3)	2159	119	2749	19
H(4)	2432	1146	2742	20
H(5)	2808	1674	4978	18
H(10)	6310	815	7911	15
H(11A)	7923	2214	14815	16
H(11B)	8650	1786	13697	16
H(14A)	1725	-250	7684	26
H(14B)	2940	285	8610	26
H(14C)	4042	-242	8016	26
H(1)	1880(30)	1512(8)	7620(20)	32(5)
H(2)	12020(30)	2854(10)	14780(30)	49(6)

Table 6. Torsion angles [°] for npr14213-lt.

C(13)-C(2)-C(3)-C(4)	0.61(19)
C(1)-C(2)-C(3)-C(4)	179.31(12)
C(2)-C(3)-C(4)-C(5)	-1.4(2)
C(3)-C(4)-C(5)-C(6)	0.1(2)
C(4)-C(5)-C(6)-C(13)	2.08(19)
C(4)-C(5)-C(6)-N(1)	-175.34(11)
C(7)-N(1)-C(6)-C(5)	-105.51(14)
C(7)-N(1)-C(6)-C(13)	77.05(16)
C(6)-N(1)-C(7)-N(2)	177.32(11)
C(6)-N(1)-C(7)-C(10)	-3.02(19)
C(8)-N(2)-C(7)-N(1)	179.68(11)
C(8)-N(2)-C(7)-C(10)	0.01(17)
C(9)-N(3)-C(8)-N(2)	-0.23(18)
C(9)-N(3)-C(8)-S(1)	179.90(9)
C(7)-N(2)-C(8)-N(3)	0.36(18)
C(7)-N(2)-C(8)-S(1)	-179.76(9)
C(11)-S(1)-C(8)-N(3)	-4.64(11)
C(11)-S(1)-C(8)-N(2)	175.46(9)
C(8)-N(3)-C(9)-C(10)	-0.31(18)
C(8)-N(3)-C(9)-Cl(1)	178.94(9)
N(3)-C(9)-C(10)-C(7)	0.63(19)
Cl(1)-C(9)-C(10)-C(7)	-178.62(9)
N(1)-C(7)-C(10)-C(9)	179.89(12)
N(2)-C(7)-C(10)-C(9)	-0.45(18)
C(8)-S(1)-C(11)-C(12)	92.29(10)
S(1)-C(11)-C(12)-O(1)	-13.22(17)
S(1)-C(11)-C(12)-O(2)	167.52(9)
C(5)-C(6)-C(13)-C(2)	-2.84(19)
N(1)-C(6)-C(13)-C(2)	174.49(11)
C(5)-C(6)-C(13)-C(14)	177.79(12)
N(1)-C(6)-C(13)-C(14)	-4.88(18)
C(3)-C(2)-C(13)-C(6)	1.47(18)
C(1)-C(2)-C(13)-C(6)	-177.23(11)
C(3)-C(2)-C(13)-C(14)	-179.14(11)

C(1)-C(2)-C(13)-C(14)	2.16(18)

Symmetry transformations used to generate equivalent atoms:

Table 7. Hydrogen bonds for npr14213-lt [Å and °].

D-H...A	d(D-H)	d(H...A)	d(D...A)	<(DHA)
C(5)-H(5)...O(2)#1	0.95	2.44	3.2803(16)	146.9
C(11)-H(11A)...O(1)#2	0.99	2.55	3.4029(16)	143.8
N(1)-H(1)...O(1)#3	0.86(2)	2.05(2)	2.9077(14)	178.6(19)
O(2)-H(2)...N(2)#4	0.85(2)	1.93(2)	2.7708(14)	168(2)

Symmetry transformations used to generate equivalent atoms:

#1 x-1,y,z-1 #2 x,-y+1/2,z+1/2 #3 x-1,-y+1/2,z-1/2

#4 x+1,-y+1/2,z+1/2

Projection view with 50% probability ellipsoids:

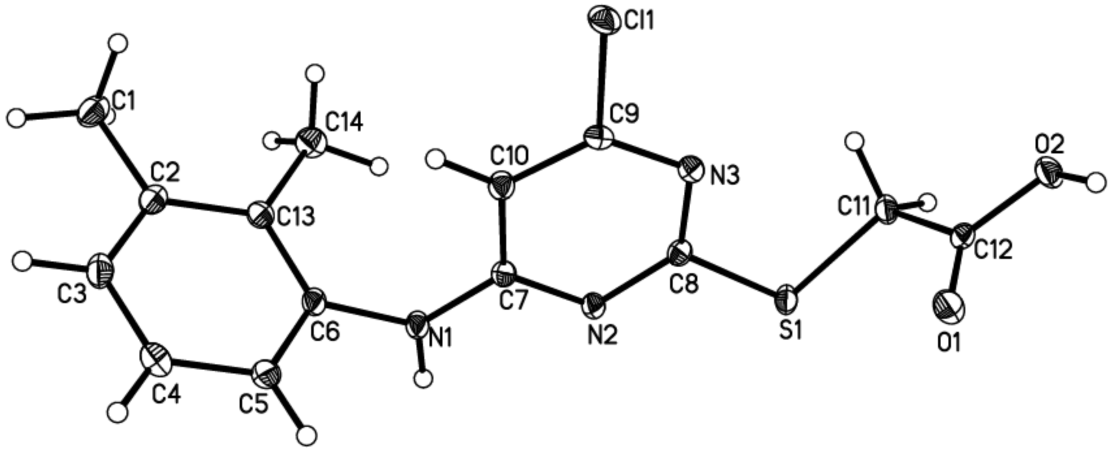

Supplement: Supplementary Information [file srep34394-s1.pdf]
